# Fourier-Transform Infrared Spectroscopy of Skeletal Muscle Tissue: Expanding Biomarkers in Primary Mitochondrial Myopathies

**DOI:** 10.3390/genes11121522

**Published:** 2020-12-19

**Authors:** Jacopo Gervasoni, Aniello Primiano, Federico Marini, Andrea Sabino, Alessandra Biancolillo, Riccardo Calvani, Anna Picca, Emanuele Marzetti, Silvia Persichilli, Andrea Urbani, Serenella Servidei, Guido Primiano

**Affiliations:** 1 Fondazione Policlinico Universitario A. Gemelli IRCCS, 00168 Rome, Italy; jacopo.gervasoni@policlinicogemelli.it (J.G.); aniello.primiano@unicatt.it (A.P.); riccardo.calvani@guest.policlinicogemelli.it (R.C.); anna.picca@guest.policlinicogemelli.it (A.P.); emanuele.marzetti@policlinicogemelli.it (E.M.); silvia.persichilli@policlinicogemelli.it (S.P.); andrea.urbani@policlinicogemelli.it (A.U.); serenella.servidei@unicatt.it (S.S.); 2 Università Cattolica del Sacro Cuore, 00168 Roma, Italy; andrea.sabino@unicatt.it; 3 Department of Chemistry, Sapienza Università di Roma, 00185 Rome, Italy; federico.marini@uniroma1.it; 4 Department of Physical and Chemical Sciences, University of L’Aquila, 67100 L’Aquila, Italy; alessandra.biancolilllo@univaq.it; 5 Aging Research Center, Department of Neurobiology, Care Sciences and Society, Karolinska Institutet and Stockholm University, 17177 Stockholm, Sweden

**Keywords:** mitochondrial diseases, FTIR, biomarkers, metabolomics, differential diagnosis, mtDNA, oculopharyngeal muscular dystrophy (OPMD), progressive external ophthalmoplegia (PEO), personalized medicine

## Abstract

Primary mitochondrial myopathies (PMM) are a group of mitochondrial disorders characterized by a predominant skeletal muscle involvement. The aim of this study was to evaluate whether the biochemical profile determined by Fourier-transform infrared (FTIR) spectroscopic technique would allow to distinguish among patients affected by progressive external ophthalmoplegia (PEO), the most common PMM presentation, oculopharyngeal muscular dystrophy (OPMD), and healthy controls. Thirty-four participants were enrolled in the study. FTIR spectroscopy was found to be a sensitive and specific diagnostic marker for PEO. In particular, FTIR spectroscopy was able to distinguish PEO patients from those affected by OPMD, even in the presence of histological findings similar to mitochondrial myopathy. At the same time, FTIR spectroscopy differentiated single mtDNA deletion and mutations in *POLG*, the most common nuclear gene associated with mitochondrial diseases, with high sensitivity and specificity. In conclusion, our data suggest that FTIR spectroscopy is a valuable biodiagnostic tool for the differential diagnosis of PEO with a high ability to also distinguish between single mtDNA deletion and mutations in *POLG* gene based on specific metabolic transitions.

## 1. Introduction

Mitochondrial diseases (MDs) are among the most common inherited metabolic disorders [1] and are characterized by a primary defect in mitochondrial oxidative phosphorylation and ATP synthesis [2]. Defects in mitochondrial DNA (mtDNA) and nuclear DNA (nDNA) are involved in the pathogenesis of MDs, resulting in a genetic complexity encompassing sporadic cases or maternal, autosomal dominant, autosomal recessive, and X-linked inheritance patterns [3]. A remarkable feature of people with primary mitochondrial dysfunction is their clinical heterogeneity, with tissues and organs with high energy demand, such as skeletal muscle, brain, and heart, being more frequently affected [4]. In particular, primary mitochondrial myopathies (PMM) are a group of mitochondrial disorders characterized by a predominant, but not exclusive, skeletal muscle involvement [5]. Progressive external ophthalmoplegia (PEO) is the most common presentation of PMM for the high reliance on oxidative metabolism of extraocular muscles. Commonly associated with a single mtDNA deletion resulting in a sporadic disease, this canonical phenotype of MD can be caused by mutations in nDNA-encoded genes implicated in mtDNA replication and maintenance and, in very rare cases, by mtDNA point mutations [5].

In the last few years, vibrational spectroscopy combined with chemometric analysis has been used in different biomedical fields, including metabolomic investigations on a variety of biological samples (i.e., tissue, cells, and other biological matrices) [6,7]. The infrared spectrum originates from the vibrational motion of the functional group of molecules. Vibrational frequencies are a sort of fingerprint of a compound and their qualities are used to characterize specific spectral signatures of the molecular content of the samples under investigation (Table 1). The main advantages of Fourier-transform infrared (FTIR) in the analysis of tissues are that it is a fast, non-destructive technique that requires small amounts of samples, and it provides information on the molecular properties of a specimen.

The aim of this study was to evaluate the ability of FTIR in characterizing the biochemical profile of muscle biopsy samples and to discriminate patients affected by PEO, including Kearns–Sayre syndrome (KSS), from those with oculopharyngeal muscular dystrophy (OPMD), a “mitochondrial disease phenocopy” with similar clinical presentations, and healthy controls.

## 2. Materials and Methods

### 2.1. Study Participants

All participants were recruited at the Fondazione Policlinico Universitario A. Gemelli IRCCS (Rome, Italy). Cases had a diagnosis of MD, with PEO phenotype, and OPMD based on histological and molecular genetics analysis.KSS was also included in the PEO group, since chronic progressive external ophthalmoplegia is the main clinical feature of this syndrome. Controls were persons who had undergone muscle biopsy for persistent hyperCKemia or myalgia in whom, however, morphology, histochemistry, and biochemistry showed no abnormalities and neurological examination was unremarkable. The study was approved by the Ethics Committee of the Università Cattolica del Sacro Cuore (Rome, Italy; ID: 1708, 21 September 2017) and all participants signed an informed consent prior to inclusion. All procedures were conducted in compliance to the ethical standards laid down in the 1964 Declaration of Helsinki and its later amendments.

### 2.2. Muscle Biopsy Collection and Fourier-Transform Infrared Measurements

A deltoid muscle specimen was collected by open biopsy under local anesthesia. Muscle samples were cleaned of any visible blood and fat, snap-frozen in liquid nitrogen, and subsequently stored at −80 °C. Specimens were examined by standard histochemical and histoenzymatic techniques. For FTIR, muscle samples were cut at 10 µm in cross-section and mounted on slides for spectral analysis. Slides were air-dried for 10 s on a Diamond/ZnSe crystal and analyzed using a Spectrum One FTIR spectrophotometer (Perkin Elmer, Norwalk, CT, USA) equipped with an attenuated total reflection (ATR) module. All FTIR spectra were recorded in the 4000–550 cm^−1^ wavenumber range, accumulating four scans at a nominal resolution of four cm^−1^. The ATR crystal was cleaned with distilled water and ethanol before every acquisition, the background was collected on an empty cell (no sample loaded) prior to any measurement and it was automatically subtracted from the spectrum of each analyzed sample. Spectra were recorded through a Spectrum v.5.0.1 PerkinElemer software (PerkinElmer, Italy) in transmittance mode and then converted to ASCII format.

### 2.3. Statistical Analyses

Descriptive statistics were used to describe demographic and clinical characteristics of the study sample. Continuous variables are reported as means and standard deviations (SDs). Categorical variables are summarized as absolute numbers and percentages.

Discrimination between cases and controls was accomplished through the use of chemometric classification methods, namely partial least squares discriminant analysis (PLS-DA) [8,9]. PLS-DA allows calculating classification models in instances where traditional linear discriminant analysis (LDA) may not be applied (e.g., spectroscopic data, where the number of measured variables largely exceeds that of available samples and predictors are highly correlated) [10]. PLS-DA is a regression-based classification technique, which implies the definition of class belonging of samples through a dummy binary response matrix and the calculation of a calibration model between the spectroscopic data set and that dummy Y, through PLS regression [11]. PLS allows overcoming the above-mentioned limitations as it operates by projecting the measurements on a lower-dimensional space spanned by so-called latent variables (components), orientated in order to to have maximum covariance with responses to be predicted. LDA can then be applied either in such a low-dimensional space or to the predicted responses to achieve classification. 

In this framework, the identification of putative wavenumber markers was carried out based on the values of the model coefficients and through the use of covariance selection (CovSel) [12]. CovSel is a parsimonious variable selection strategy which identifies the least redundant set of measured variables having maximum covariance with the responses. In all cases, the predictive outcomes of the models were validated using repeated double cross-validation (rDCV) [13,14]. The latter allows obtaining unbiased estimates of the predictive ability of a chemometric/statistical model as it is based on dividing the samples into two intertwined loops of cross-validation: the inner loop for model selection and the outer loop for the actual validation of the predictions of the model built on inner loop samples. A more detailed description of multivariate analytical strategies can be found elsewhere [9].

## 3. Results

Thirty-four participants, 18 females (52.9%) and 16 males (47.1%), were enrolled in the study. The sample included 16 participants with PEO (8 associated with single mtDNA deletion, including 3 KSS; 8 with mutations in *POLG*), nine with OPMD with mutations in *PABPN1*, and nine controls without histological or clinical evidence of myopathy. The mean age at the time of muscle biopsy was 44.2 years (SD: 17.7), with a range of 11–80 years. Deltoid muscle biopsies showed consistent mitochondrial abnormalities in three out of nine participants with OPMD, with classic ragged-blue fibers (RBF) pale at the cytochrome c oxidase (COX) stain. The main characteristics of the 25 participants with PEO or OPMD are summarized in Table 2.

We first explored the possibility of using FTIR spectroscopy to differentiate muscle biopsies obtained from PEO, OPMD, and control participants. To this purpose, a PLS-DA model was built on mean-centered data and validated by rDCV with 50 runs involving 12 and eight cancelation groups in the outer and inner loop, respectively (Table 3).

**Figure 1:**
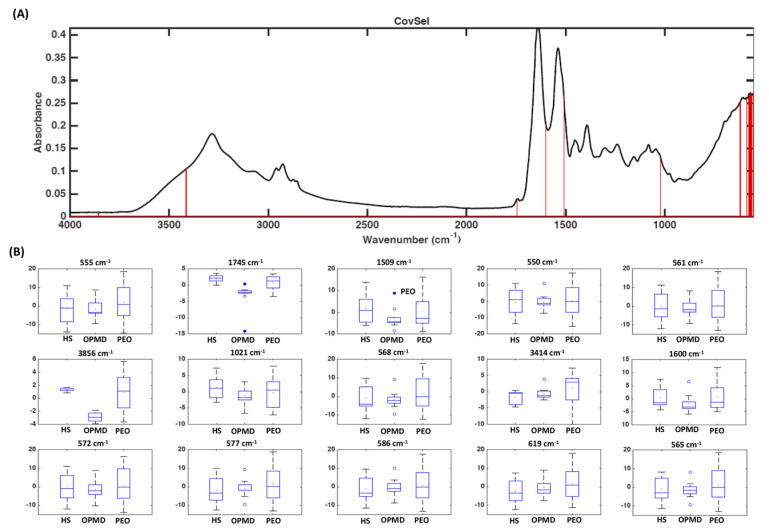
Fourier-transform infrared spectra recorded in muscle samples from participants with progressive external ophthalmoplegia (PEO), oculopharyngeal muscular dystrophy (OPMD), and controls (HS). (**A**) Covariance selection (CovSel) analysis: representation of the variables having a significant value (red bars) overlapped to the average infrared spectrum (black line); (**B**) boxplot of discriminant wavenumbers.

The results obtained show that the spectroscopic fingerprint embeds sufficient information to allow correct classification of the majority of participants, who are in turn left out as validation individuals. The same classification rates were obtained when selecting the minimum pool of discriminant variables, which could be considered as putative markers, by the CovSel algorithm. In particular, it was found that only few wavenumbers (3856 cm^−1^, 3414 cm^−1^, 1745 cm^−1^, 1600 cm^−1^, 1509 cm^−1^, 1021 cm^−1^, 619 cm^−1^, 586 cm^−1^, 577 cm^−1^, 572 cm^−1^, 568 cm^−1^, 565 cm^−1^, 561 cm^−1^, 555 cm^−1^, 550 cm^−1^) were sufficient to achieve the same predictive ability as with the full spectral data (Figure 1).

In a second stage, the classification analysis was repeated on the reduced data set including only measurements collected from participants with PEO, in order to verify whether it could be possible to discriminate single mtDNA deletion from *POLG* mutations. The PLS-DA model was built and validated similarly to what has been already described. The results are summarized in Table 4 and indicate that the infrared fingerprint is informative enough to provide accurate discrimination between PEO participants with single mtDNA deletion and those with *POLG* mutations. The classification accuracy was almost 100%, highlighting the potential value of the proposed approach.

**Figure 2:**
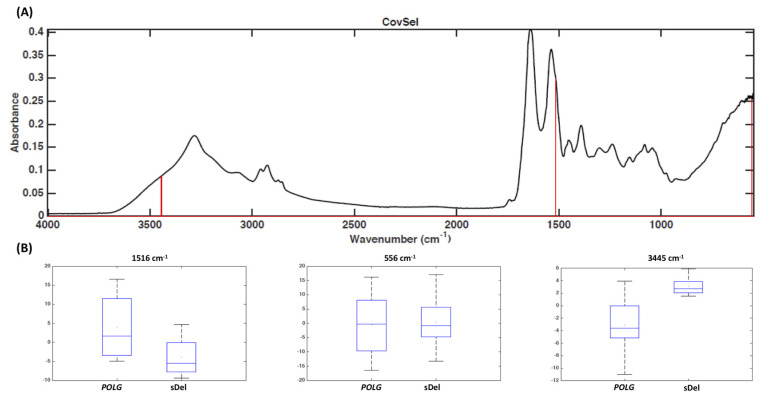
Fourier-transform infrared spectra recorded in muscle samples from participants with progressive external ophthalmoplegia with *POLG* mutations or single mitochondrial DNA deletion (sDel). (**A**) Covariance selection (CovSel) analysis: representation of the variables having a significant value (red bars) overlapped to the average infrared spectrum (black line); (**B**) boxplot of discriminant wavenumbers.

Variable selection by means of the CovSel algorithm indicated that the same classification accuracy in prediction could be achieved when retaining only three wavenumbers, namely 556 cm^−1^, 1516 cm^−1^, and 3445 cm^−1^ (Figure 2), which can therefore be considered putative markers for discrimination.

## 4. Discussion

The detection of a single mtDNA deletion in patients with mitochondrial myopathy described by Holt et al. in 1988 [15] inaugurated the molecular era of mitochondrial medicine. Other research groups that associated single mtDNA deletions with KSS [16] and sporadic PEO [17] confirmed these findings. Immediately afterwards, Zeviani et al. [18] described several Italian patients with PEO and multiple mtDNA deletions characterized by autosomal dominant or recessive mitochondrial PEO, demonstrating a dual genetic control of mitochondrial function with mtDNA and nDNA working in concert. In addition to their genetic complexity, MDs are a diagnostic challenge for clinical overlap between the classic mitochondrial syndrome and other inherited diseases, such as OPMD.

In the present study, we have shown that the biochemical profile of muscle samples determined by FTIR spectroscopic technique is a sensitive and specific diagnostic marker for PEO, which represents the most common presentation of PMM. In particular, FTIR spectroscopy coupled with chemometric analysis was able to distinguish PEO patients from those affected by OPMD and mutations in *PABPN1*. This is particularly interesting for the following reasons: (1) OPMD can be considered a “mitochondrial disease phenocopy” for its clinical similarities with PEO; (2) the presence of histological overlap between mitochondrial myopathies and OPMD (33% of patients with mutations in *PABPN1* showed histological evidence of mitochondrial pathology); (3) it is not possible to detect triplet repeat expansion in *PABPN1* with a next-generation sequencing technique. Although no effective treatments are currently available for PEO or OPMD, a correct genetic diagnosis is instrumental for MDs that require specific clinical or pharmacological management [19,20,21]. At the same time, FTIR spectroscopy differentiated with a high sensitivity and specificity PEO participants with single mtDNA deletion from those with mutations in *POLG*, the most common nuclear gene associated with PEO in an Italian cohort of patients with MD [22]. This information has relevant implications concerning the risk of inheriting the disease. Overall, our data show that the biochemical profile of skeletal muscle obtained by FTIR spectroscopy coupled with chemometric analysis has good sensitivity and specificity for detection of patients with PEO and for discrimination between single mtDNA deletion and mutations in *POLG* (Table 3 and Table 4). Moreover, the discriminant spectroscopic transitions are suggestive of molecular species which are in line with the expected metabolic phenotype of the investigated syndromes. For all these reasons, in an ideally diagnostic algorithm for the investigation of patients with ptosis and/or limitations of eye movements, FTIR spectroscopy could precede Southern blot or long-range PCR for its high ability to differentiate single mtDNA deletion from mutations in nuclear genes, such as *POLG* or *PABPN1*. A further advantage of FTIR compared with traditional techniques is the smaller amount of muscle needed. Only few wavenumbers were sufficient to achieve the same predictive ability as with the full spectral data (Figure 1 and Figure 2). The difference between *POLG* mutations and single mtDNA deletion in the absorption band of proteins (Figure 2) might reflect distinct pathophysiological mechanisms between the two types of genetic abnormalities. 

Future directions will include the evaluation of the ability of FTIR spectral signatures to discriminate among different genotypes of MDs by testing skeletal muscle samples from a larger cohort of patients. These studies will also allow establishing whether age and other patient characteristics affect FTIR signatures. The value of FTIR metabolic profiles as prognostic biomarkers in MDs is also worth exploration. Finally, studies are needed to evaluate and validate FTIR spectrum derived from biofluids (e.g., blood and urine) as a non-invasive biomarker of PMM and other MDs.

## Figures and Tables

**Table 1:** Typical absorption bands acknowledged in tissues using spectrum in the wavenumber range 4000–400 cm^−1^.

Spectral Region (cm^−1^)	Biomolecular Peak Assignments
3000–2800	Symmetric and asymmetric C-H stretching vibrations of lipids
1745–1725	Symmetric C=O stretching vibration of lipids
1700–1600	Amide I region of proteins carrying information on secondary structure
1600–1500	Amide II region of proteins
1200–900	Symmetric and asymmetric PO_3_^−^ stretching vibration and symmetric CO-O-C stretching vibration of nucleic acids and carbohydrates

**Table 2:** Demographic, histopathological, and genetic characteristics of participants with progressive external ophthalmoplegia (PEO) or oculopharyngeal muscular dystrophy (OPMD).

Participants	Sex	Age at Biopsy (Years)	Phenotype	Genotype
1	M	27	PEO	c.2864A>G [p.Y955C] *POLG*
2	F	64	PEO	c.2864A>G [p.Y955C] *POLG*
3	F	47	PEO	c.1943C>G [P648R]/c.2243G>C [p.W748S] *POLG*
4	F	52	PEO	c.926G>A [p.R309H]/c.1760C>T [p.P587L] *POLG*
5	F	61	PEO	c.3293A>G [p.1098N>S] *POLG*
6	M	44	PEO	c.2864A>G [p.Y955C] *POLG*
7	F	39	PEO	c.2864A>G [p.Y955C] *POLG*
8	F	35	PEO	c.2864A>G [p.Y955C] *POLG*
9	M	14	PEO	single mtDNA deletion
10	F	31	PEO	single mtDNA deletion
11	F	31	PEO	single mtDNA deletion
12	F	62	PEO	single mtDNA deletion
13	M	54	PEO	single mtDNA deletion
14	M	13	PEO	single mtDNA deletion
15	M	11	PEO	single mtDNA deletion
16	F	23	PEO	single mtDNA deletion
17	M	62	OPMD	*PABPN1* (GCN)13/Ala13
18	F	55	OPMD	*PABPN1* (GCN)13/Ala13
19	M	56	OPMD	*PABPN1* (GCN)15/Ala15
20	M	54	OPMD	*PABPN1* (GCN)15/Ala15
21	M	62	OPMD	*PABPN1* (GCN)15/Ala15
22	F	61	OPMD	*PABPN1* (GCN)13/Ala13
23	F	71	OPMD	*PABPN1* (GCN)12/Ala12
24	F	80	OPMD	*PABPN1* (GCN)11/Ala11
25	M	59	OPMD	*PABPN1* (GCN)13/Ala13

**Table 3:** Results of partial least squares discriminant analysis modeling of participants with progressive external ophthalmoplegia (PEO), oculopharyngeal muscular dystrophy (OPMD), and controls (outer loop).

Class	Sensitivity	Specificity	Accuracy	Average Classification Error
PEO	85.7 ± 7.2%	94.7 ± 3.7%	90.1 ± 4.2%	8.7 ± 3.8%
OPMD	96.4 ± 5.2%	95.7 ± 4.0%		
Controls	91.7 ± 6.9%	94.7 ± 2.6%		

**Table 4:** Results of partial least squares discriminant analysis modeling of participants with progressive external ophthalmoplegia (outer loop).

Class	Sensitivity	Specificity	Accuracy	Average Classification Error
Single mtDNA deletion	100.0 ± 0.0%	99.0 ± 3.4%	99.5 ± 1.7%	0.5 ± 1.7%
*POLG* mutations	99.0 ± 3.4%	100.0 ± 0.0%		
